# Ultrasound imaging of the femoral and saphenous nerves

**DOI:** 10.1002/ajum.12403

**Published:** 2024-07-29

**Authors:** Michelle Fenech, Bridie Roche, Jerome Boyle

**Affiliations:** ^1^ School of Health, Medical and Applied Sciences, College of Clinical Sciences Central Queensland University Brisbane Queensland Australia; ^2^ Department of Medical Imaging Royal Brisbane and Women's Hospital Herston, Brisbane Queensland Australia; ^3^ Imaging Associates Group Box Hill Victoria Australia

**Keywords:** femoral nerve, lower limb nerves, musculoskeletal ultrasound, saphenous nerve, sonography

## Abstract

**Introduction:**

Iatrogenic and traumatic injuries to the femoral and saphenous nerves, and their branches are uncommon but can be a cause of clinically pertinent lower limb dysfunction and neuralgia. Despite this, direct sonographic imaging of these nerves is not commonly requested or performed.

**Methods:**

A review of the literature regarding the detailed relative anatomy, sonographic technique to image these nerves and their branches and their normal and abnormal appearances was conducted.

**Discussion:**

These nerves are often in the direct imaging field of many ultrasound examinations including the assessment of the groin and lower limb vasculature and musculoskeletal studies. They can become entrapped at certain points throughout their path, where particular attention should be provided to these nerves.

**Conclusion:**

Improved knowledge regarding the sonographic imaging of the femoral and saphenous nerves and their branches can assist identification and discrimination between normal and abnormal appearances, and subsequent ultrasound‐guided nerve blockades or radiofrequency ablations for pain management where required.

## Introduction

Sonographically guided femoral and saphenous nerve blockades or radiofrequency ablations can be used as interventions for pain management.^1^ The success of these procedures relies on knowledge of the neural sono‐anatomy, safe planning of the needle path and needle placement as close to the nerve as possible without direct contact. Femoral nerve (FN) blocks can be used for pain control post‐femoral fractures and post‐hip fracture surgery.^2^ Saphenous neuralgia describes pain in the distribution of the saphenous nerve, the longest branch of the FN. Saphenous nerve blocks can be used for pre‐ and post‐operative anaesthesia and for analgesia in suspected nerve entrapments. The infrapatellar branch of the saphenous nerve can be targeted with nerve blocks to address knee pain after knee surgery or trauma.^3^ Despite the femoral and saphenous nerves being within the field of view during many routine sonographic examinations, these nerves are frequently overlooked and underappreciated during groin, lower limb venous and arterial studies and musculoskeletal examinations.

## Anatomy and sonographic imaging of the femoral nerve

**Figure 1:**
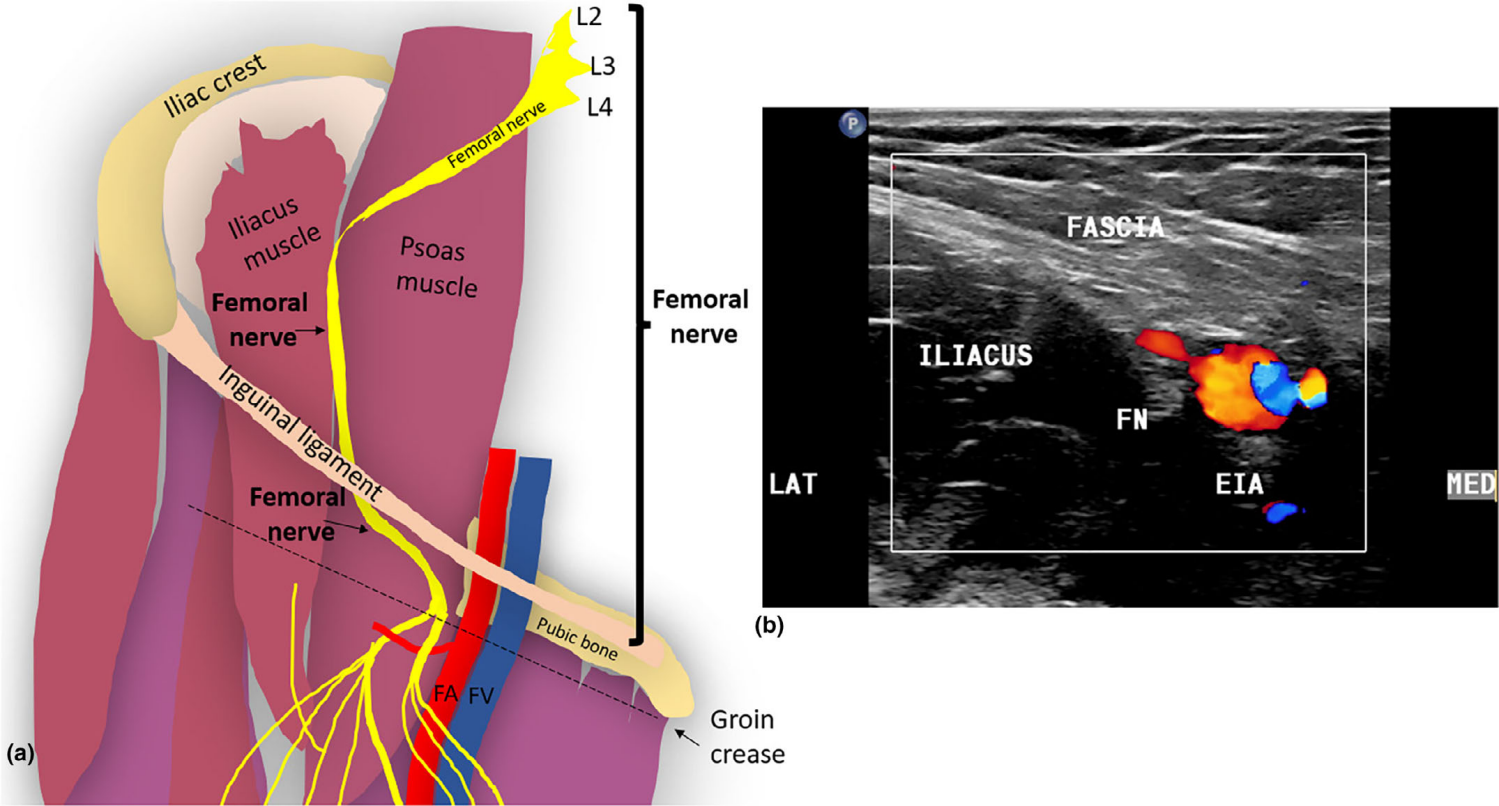
(a) Course of the right femoral nerve through the pelvis. (b) Transverse sonographic image of the right femoral nerve (FN), deep to the fascia iliaca, lateral to the external iliac artery (EIA). FA, femoral artery; FV, femoral vein; LAT, lateral aspect of the image; MED, medial aspect of the image.

The FN is a mixed motor and sensory nerve and the largest branch of the lumbar plexus. It originates from the posterior divisions of the anterior rami of nerve roots L2–L4.^4^ The FN then descends through the psoas major muscle with a medial to lateral trajectory.^5^ It exits the lateral border of the psoas major muscle, about 4 cm superior to the inguinal ligament, and passes between the psoas major and iliacus muscles, deep into the iliac fascia.^6^ Superior to the level of the inguinal ligament, the FN gives rise to variable motor branches that provide innervation for the iliacus and pectineus muscles.^5^ Superior to the inguinal ligament, the FN and its branches can be challenging to identify sonographically, owing to their deep location. The FN courses into the groin and proximal thigh, deep to the inguinal ligament, to lie lateral to the femoral artery (FA) in the femoral triangle^7^ (Figure 1).

### Femoral nerve at the level of the inguinal ligament

**Figure 2:**
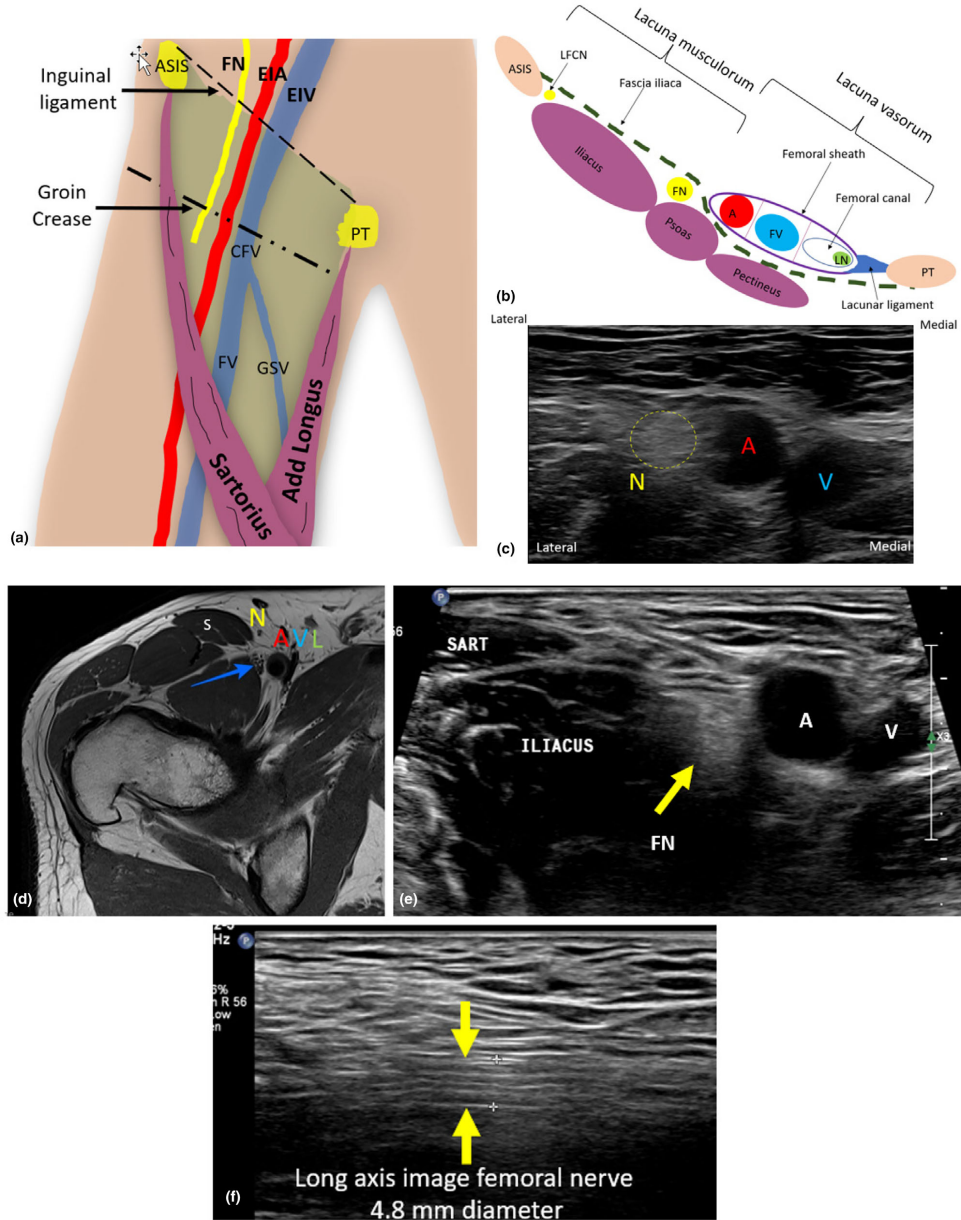
Relative position of the right femoral nerve (FN). (a) The inguinal ligament extends between the anterior superior iliac spine (ASIS) and pubic tubercle (PT). At this level, the FN sits lateral to the external iliac artery (EIA) and external iliac vein (EIV). The inguinal ligament forms the superior border of the femoral triangle (green), which is outlined by the sartorius and adductor longus muscles. (b) Cross‐sectional view of the groin, demonstrating the femoral sheath and femoral canal medially, which contain a potential space, and the lymph node of Cloquet (LN). The FN and lateral femoral cutaneous nerve (LFCN) both lie deep to the fascia iliaca. (c) A sonographic short‐axis image of the right groin, demonstrating the lateral‐to‐medial relationship of the femoral nerve (N) outlined by yellow dotted line, artery (A) and vein (V). (d) Axial MRI demonstrating the relationship between the FN (blue arrow) and the sartorius muscle (S) lateral to it. (e) Short‐axis image of the femoral nerve (yellow arrow) just superior to the groin crease, demonstrating the sartorius muscle lateral to the FN. (e, f) Long‐axis sonographic image of the FN (outlined between yellow arrows). A, artery; CFV, common femoral vein; FV, femoral vein; GSV, great saphenous vein; L, region of lymphatic channels; N, femoral nerve; SART; sartorius muscle; V, vein.

The inguinal ligament forms the superior border of the femoral triangle and is located approximately 2 cm superior to the groin crease.^8^ The femoral triangle, also called Scarpa's triangle, is a large triangular space in the proximal anterior thigh, demarcated infero‐laterally by the sartorius muscle and infero‐medially by the adductor longus muscle.^6^ The sartorius muscle is an important landmark for sonographic identification of the FN, its divisions and the saphenous nerve in the thigh. At the level of the inguinal ligament, the FN can be sonographically demonstrated in the short axis, immediately lateral to the external iliac artery (EIA) and vein; it will appear echogenic and ellipsoid in shape, with internal hypoechoic rounded fascicles. Sonographic visibility of the FN in the short axis is dependent on the transducer angulation and subsequent angle of insonation. Failure to achieve a perpendicular angle of insonation may make the nerve isoechoic to the adjacent iliopsoas apparatus and therefore difficult to visualise. Colour Doppler can help to distinguish the more medially located EIA and common femoral artery (CFA) from the FN (Figure 2).

The iliopectineal arch is a vertical band that divides the region subjacent to the inguinal ligament into a lateral compartment called the ‘lacuna musculorum’, and a medial compartment called the ‘lacuna vasorum’.^9^ The medial compartment houses the external iliac and femoral vessels, which are encircled by the femoral sheath. At its proximal extent, the femoral sheath coalesces with the transversalis fascia anteriorly and posteriorly with the fascia iliaca. The sheath extends approximately 2 cm inferior to the groin crease.^6^ The FN is located outside and lateral to the funnel‐shaped femoral sheath. It is housed within the lateral compartment along with the iliopsoas muscles and tendon, and more laterally, the lateral femoral cutaneous nerve.^9^

The femoral sheath is divided into three compartments by a vertical septa of connective tissue, separating it into distinct functional areas from lateral to medial.^6^ The lateral compartment contains the EIA/CFA/Femoral artery (FA) and the femoral branch of the genitofemoral nerve.^10^ The intermediate compartment contains the following veins: external iliac vein (EIV), common femoral vein (CFV) and femoral vein (FV). The most medial and smallest compartment, the femoral canal, contains fibrofatty tissue, a singular lymph node (the node of Cloquet) and a potential space allowing for the expansion of the veins and lymphatic vessels.^6^ The femoral ring is the superior opening of the sheath into the abdominal cavity and is the site of origin for femoral hernias.^10^

Owing to the intimate relationship of the FN to the surrounding anatomy, neural compromise can occur, leading to femoral neuralgia. FN compression, injury, or lateral displacement, which can cause the nerve to be stretched, can occur at different levels, including at the lumbar roots, between the psoas and iliacus muscles, where the overlying fascia is thicker, deep to the inguinal ligament, at the hip joint and pubic bone levels (groin crease).^11^ Compression or lateral displacement of the FN can cause a confusing clinical picture of hip, groin and neural symptoms. Pathological entities of the iliopsoas musculature, abscesses, marked distension of the iliopsoas bursa, acetabular ganglia, pseudoaneurysm of the FA, or acute compartment syndrome from pelvic fractures, or fractures themselves can cause compression, irritation, injury and/or displacement of the FN. Iatrogenic FN injury can also occur due to gynaecological surgery, hip replacement procedures and fixations for pelvic and hip fractures.^9^ Ultrasound imaging can play a role in detecting direct causes of compression or injury to the FN in the groin and proximal thigh region.

### Anterior and posterior divisions of the femoral nerve

**Figure 3:**
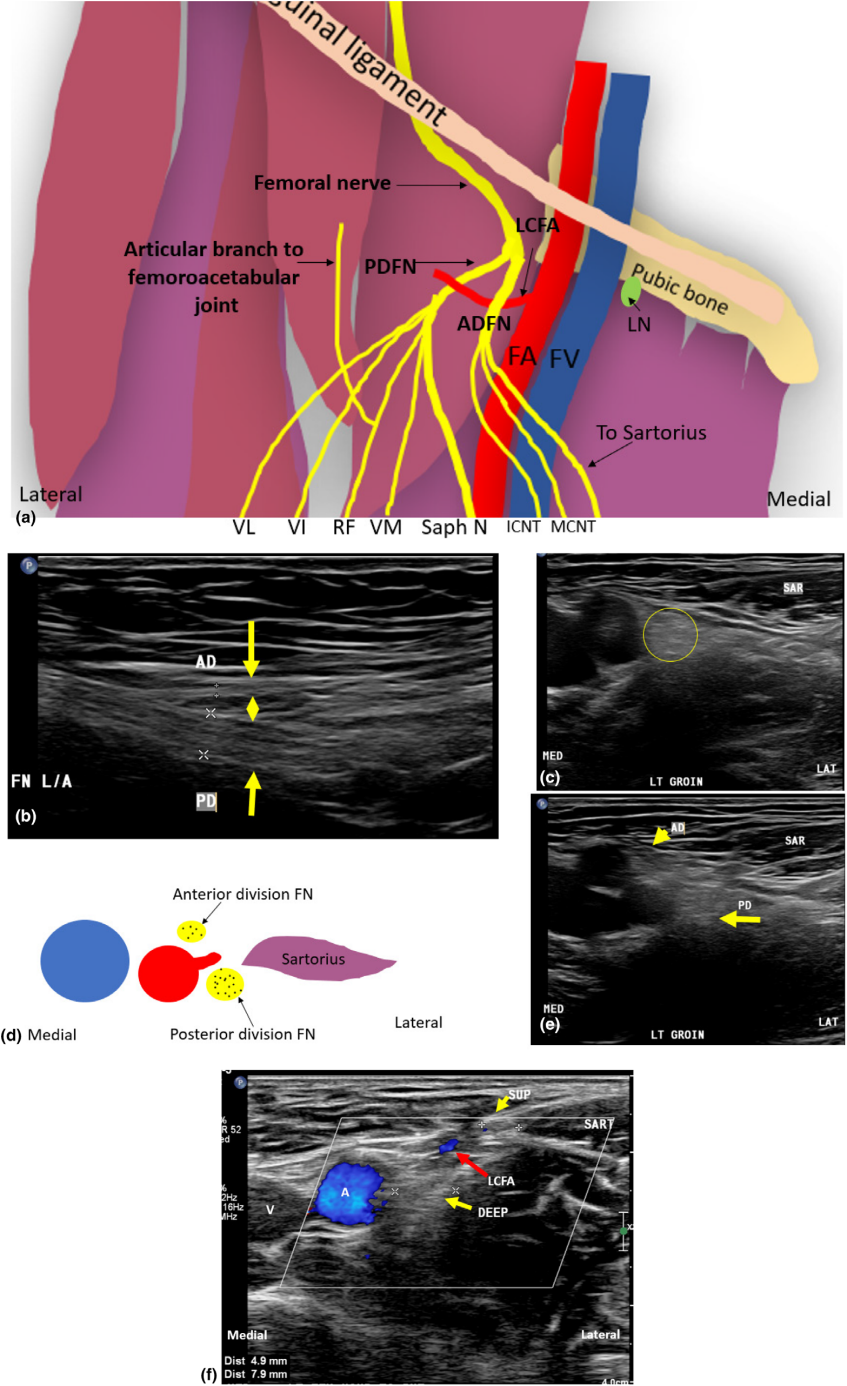
Divisions and branching of the left femoral nerve (FN). (a) The FN divides into anterior and posterior divisions, which are separated by the lateral circumflex femoral artery (LCFA). The posterior division FN gives rise to motor branches to the vastus medialis (VM), rectus femoris (RF), vastus intermedius (VI) and vastus lateralis (VL), and an articular branch (b) to supply the hip joint as well as the saphenous nerve. (b) A long‐axis sonographic image of the FN and its anterior and posterior divisions is outlined by yellow arrows. (c) A short‐axis view of the left femoral nerve (outlined by a yellow circle) lateral to the artery. (d) Diagram showing the divisions of the FN. (e) A short‐axis sonographic image demonstrating the FN anterior and posterior divisions (outlined by yellow arrows) medial to the sartorius muscle (SAR). (f) Short‐axis view with colour Doppler outlining the LCFA (red arrow) between the anterior (superficial; SUP) and posterior (deep) divisions of the FN (outlined by yellow arrows), moving to sit superficial and deep to the medial border of the sartorius muscle (SART). AD, anterior division femoral nerve; ADFN, anterior division femoral nerve; FA, femoral artery; FN, femoral nerve; FV, femoral vein; ICNT, intermediate cutaneous nerve of the thigh; L/A, long axis image; LAT, lateral aspect of image; LN, lymph node of Cloquet (green); MCNT, medial cutaneous nerve of the thigh; MED, medial aspect of image; PD, posterior division femoral nerve; PDFN, posterior division femoral nerve; Saph n, saphenous nerve.

The FN bifurcates into anterior and posterior divisions approximately 1–4 cm inferior to the inguinal ligament.^4^ These two divisions are often separated by the lateral circumflex femoral artery, a branch of the profunda FA.^6^, ^12^, ^13^ While variations of the FN branching patterns are not infrequent,^5^, ^14^ the anterior and posterior FN divisions are more consistent and can be easily appreciated sonographically^13^ (Figure 3).

#### Anterior division of the femoral nerve

The anterior division of the femoral nerve (ADFN) gives rise to sensory branches and, variably, a motor branch to innervate the sartorius muscle.^5^ The ADFN courses distally towards the superficial aspect of the sartorius muscle. Iatrogenic injury to ADFN and its branches can occur secondary to surgery or interventional procedures involving the FA.^15^ The proximal portion of the ADFN can be identified sonographically in the short axis at the level of the groin crease, between the medially located FA and the laterally positioned sartorius muscle.^16^ The ADFN sensory branches are responsible for the sensory innervation of the anteromedial thigh.^6^ They include the medial cutaneous nerve of the thigh and the intermediate cutaneous nerve of the thigh.^16^ These branches may be sonographically demonstrated to travel within subcutaneous fat overlying the sartorius muscle proximally and the vastus medialis and rectus femoris muscles as they move distally.^16^

#### Posterior division of the femoral nerve

The posterior division of the femoral nerve (PDFN) is the larger, deeper division of the FN. After arising from the FN, it passes deep to the medial border of the sartorius muscle.^16^ The PDFN typically gives rise to six branches; four motor branches; an articular branch; and a sensory branch.^11^ The four motor branches innervate the muscles of the quadriceps femoris and include: (1) the nerve to the vastus medialis (NVM), (2) the nerve to the vastus lateralis (NVL), (3) the nerve to the vastus intermedius (NVI) and (4) the nerve to the rectus femoris (NRF).^13^ The articular branch of the PDFN travels towards the femoroacetabular joint. The saphenous nerve is the sensory branch of the PDFN, the longest cutaneous branch of the FN, and is most often targeted for post‐operative analgesia following knee arthroplasty or anterior cruciate ligament repair.^7^

## Anatomy and sonographic imaging of the saphenous nerve

**Figure 4:**
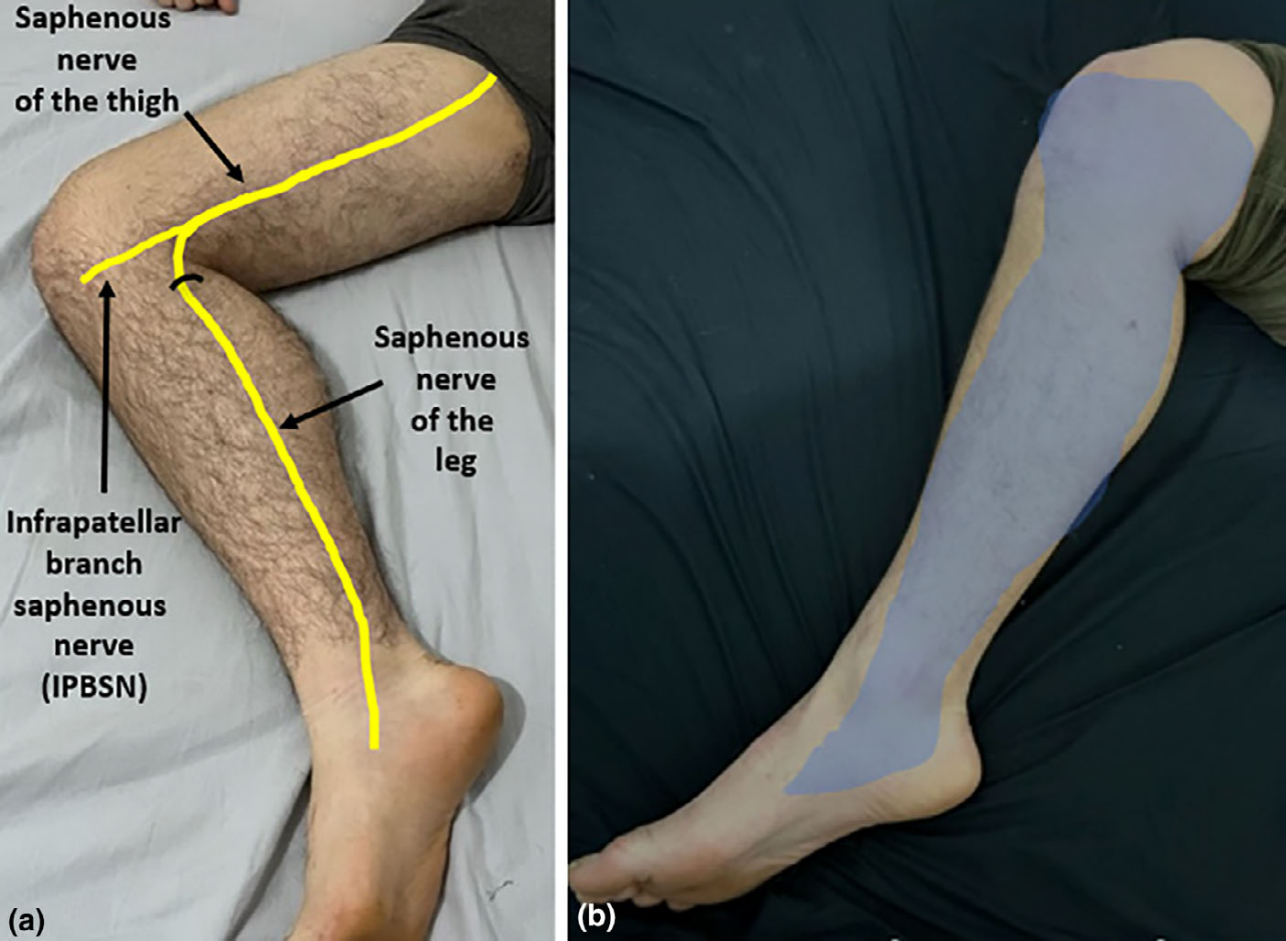
(a) Course of the saphenous nerve through the leg. At the distal thigh, it gives rise to the infrapatellar branch of the saphenous nerve. The saphenous nerve continues distally to the lower leg. It becomes superficial in location at a variable point between the distal thigh and proximal lower leg (black semicircle), emerging between the distal sartorius and gracilis muscles. (b) Area of sensory innervation of the saphenous nerve (highlighted in blue).

The saphenous nerve provides sensory innervation to the medial, anteromedial and posteromedial aspects of the lower limb.^16^, ^17^ This includes the distal thigh, pre‐patellar region, medial calf and medial arch of the foot.^18^ The saphenous nerve at the distal thigh gives rise to an infrapatellar branch and then continues distally to the lower leg, where it can sometimes be termed the ‘sartorial branch’ of the saphenous nerve (Figure 4).

### Saphenous nerve in the adductor canal of the thigh

**Figure 5:**
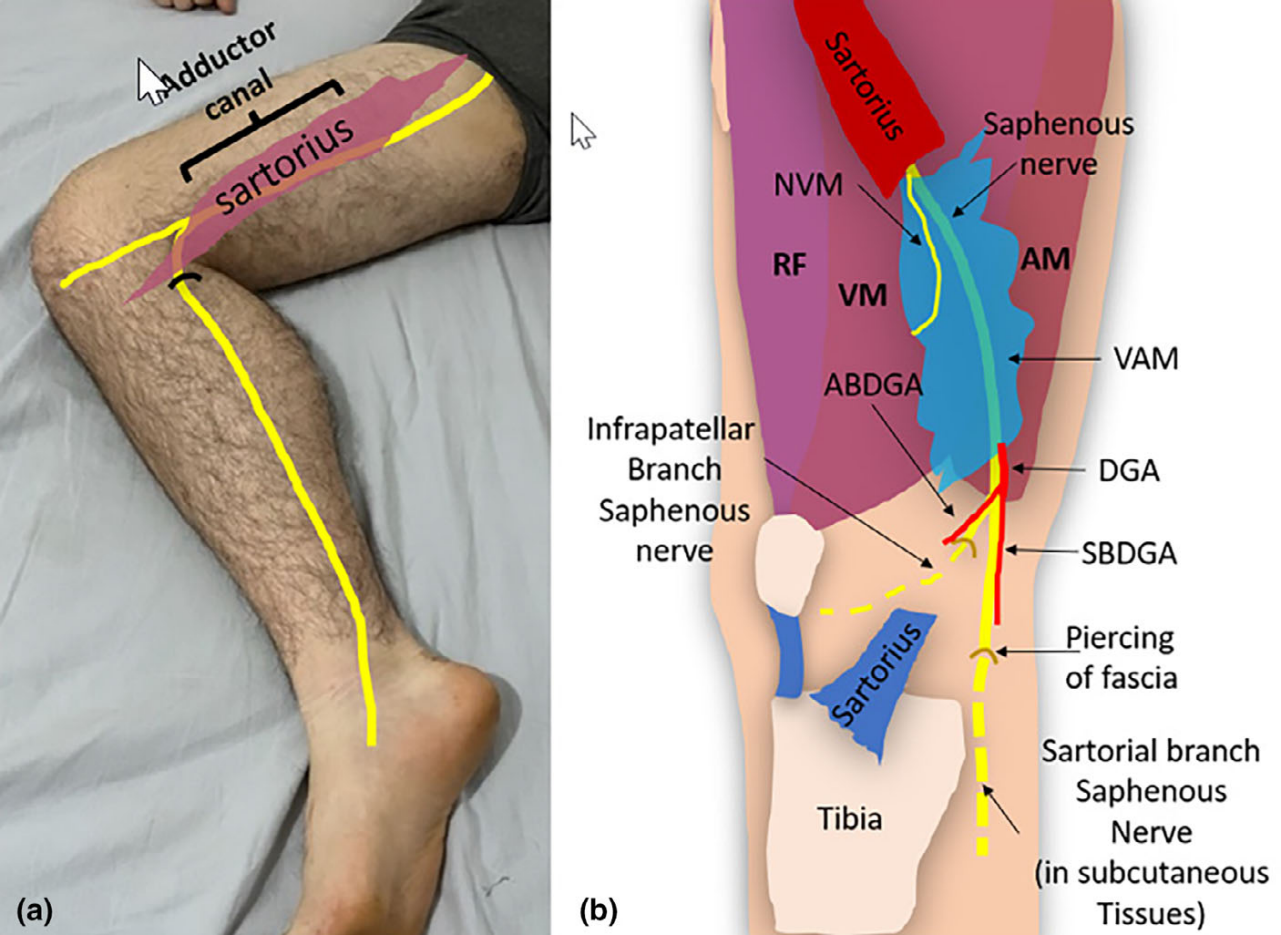
The saphenous nerve in the thigh. (a) Course of the saphenous nerve through the thigh and its sub‐sartorial section. The surface anatomy outlines the adductor canal. Black semicircle indicates where nerve moves from deep to superficial. (b) Anatomy diagram of the saphenous nerve in the adductor canal, deep to the vasto‐adductor membrane (VAM). The nerve to the vastus medialis (NVM) is located within the adductor canal but sits anterior to the saphenous nerve and superficial to the VAM. ABDGA, articular branch of the DGA; AM, adductor magnus muscle; DGA, descending genicular artery; RF, rectus femoris muscle; SBDGA, saphenous branch of DGA; VM, vastus medialis muscle.

After separating from the FN in the proximal thigh, the saphenous nerve courses medially in the upper thigh, positioned laterally in relation to the femoral vessels.^4^ The saphenous nerve then enters the adductor canal, a musculoaponeurotic tunnel of variable length (patient height dependent), also called the sub‐sartorial or Hunter canal.^19^ The adductor canal extends from the apex (inferior portion) of the femoral triangle, at the intersection of the sartorius and adductor longus muscles, to the adductor hiatus.^19^, ^20^ The adductor canal has a pertinent anatomical function: to allow passage of the FA, vein and branches of the FN, including the saphenous nerve.^21^, ^22^ The NVM, which contributes to sensory innervation of the anteromedial knee through genicular nerves, can be identified sonographically along with the saphenous nerve in the proximal half of the adductor canal.^6^, ^23^, ^24^ Nerve block interventions to the saphenous nerve for pain management can occur at this sub‐sartorial location, more commonly in the region of the distal half of the adductor canal (Figure 5).

**Figure 6:**
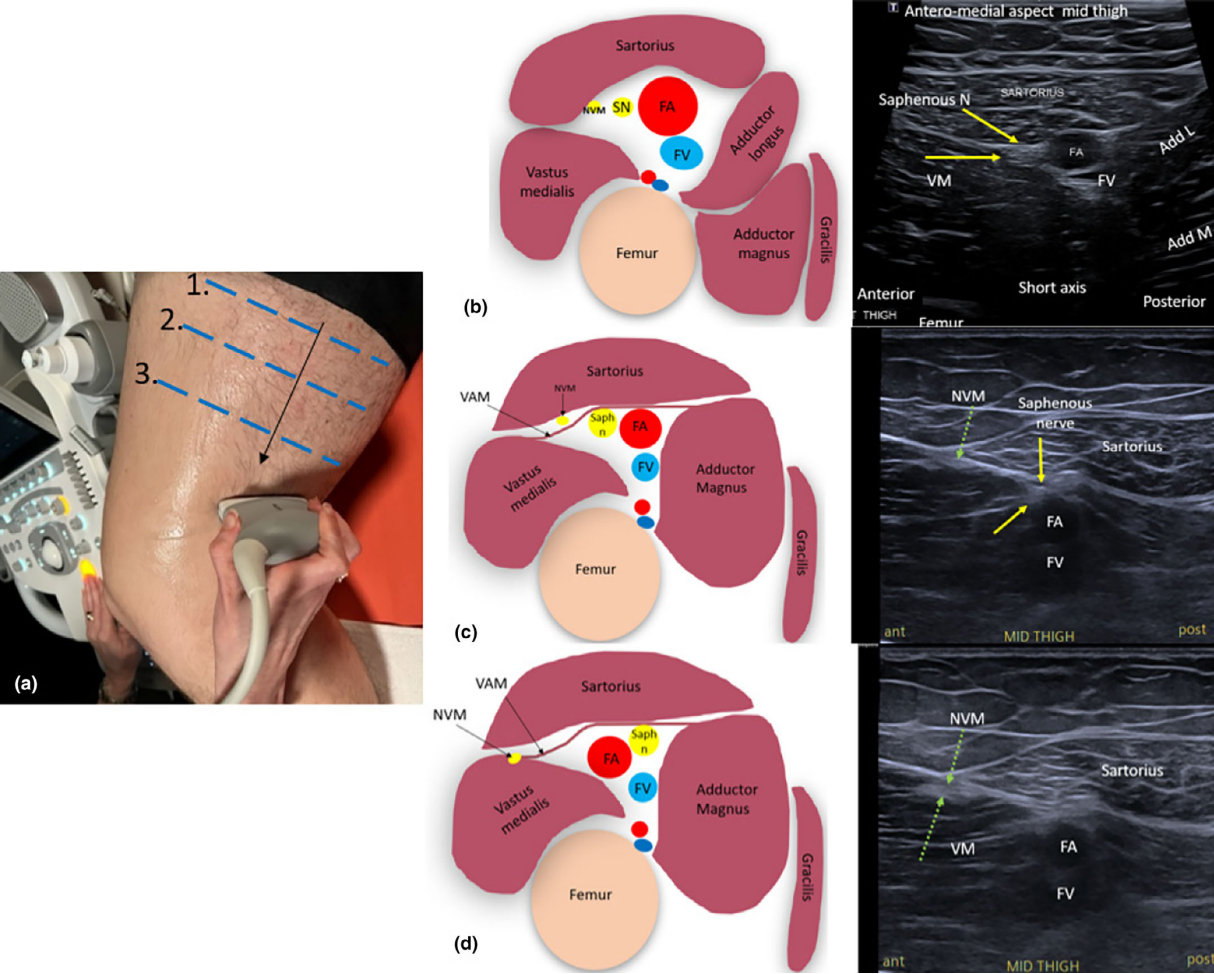
Sonographic imaging and anatomy of the saphenous nerve (Saph n) and nerve to the vastus medialis (NVM) within the adductor canal at three locations from proximal to distal. (a) Photo indicating transducer positions for short‐axis imaging of the saphenous nerve within the adductor canal. Figures (b), (c) and (d) correspond to transducer positions 1, 2 and 3 (blue lines) in the photo in (a). The NVM (green dashed arrows) is located anterior to the saphenous nerve and superficial to the vasto‐adductor membrane (VAM). On axial sonographic sweeps from proximal to distal, the saphenous nerve (yellow arrows) lies deep to the sartorius muscle and appears to cross superficially to the femoral artery (FA) in an anterior to posterior direction. Add L, adductor longus muscle; Add M, adductor magnus muscle; ant, anterior aspect of image; FA, femoral artery; FV, femoral vein; post, posterior aspect of image; SN, saphenous nerve; VM, vastus medialis muscle.

The musculoaponeurotic structures that form the borders of the adductor canal and the canal contents can readily be identified with ultrasound. The roof (or anteromedial border) of the adductor canal is formed by the sartorius muscle, which will be seen to overlie the femoral vessels and saphenous nerve when using a medial acoustic window of the mid‐ to distal thigh.^16^ This usually requires slight flexion of the knee and external rotation of the hip. The antero‐lateral border of the canal is formed by the vastus medialis muscle. The postero‐medial border of the adductor canal is formed by the adductor longus and magnus muscles. The sartorius muscle and FA are good sonographic landmarks to use to identify the saphenous nerve in the thigh. In a short‐axis sonographic sweep of the adductor canal from proximal to distal, the saphenous nerve will be observed to cross over the FA in an anterolateral to posteromedial direction.^25^ Just proximal to the adductor hiatus, the saphenous nerve separates from the femoral vessels. The nerve will maintain its position in the medial distal thigh, while the vessels will course posteriorly towards the popliteal fossa^3^ (Figure 6).

The vasto‐adductor membrane (VAM), also called the medial intermuscular septum, connects the medial edge of the vastus medialis muscle to the lateral edge of the adductor magnus muscle throughout the adductor canal.^22^ It creates superficial and deep sub‐compartments within the adductor canal. The saphenous nerve is located deep to the VAM within the deep sub‐compartment. The NVM sits in the superficial sub‐compartment, between the VAM and the sub‐sartorial fascia, anterior to the femoral vessels. The NVM, when followed distally on a short axis sonographic sweep, when imaging the canal from the medial aspect of the thigh, will appear to travel superficially and anteriorly relative to the path of the saphenous nerve and femoral vessels. At the level of the mid‐canal, the NVM can be observed sonographically to leave the adductor canal and pass into the vastus medialis muscle where it innervates.^21^

### Saphenous nerve at the adductor hiatus

The adductor hiatus (distal end of the adductor canal) is located between the adductor magnus muscle and the femur. The femoral vessels exit the adductor canal, and become anatomically considered popliteal vessels.^4^ As the FA exits the adductor canal, it gives rise to the descending genicular artery (DGA). The DGA subsequently trifurcates into a muscular branch, an articular branch and a saphenous branch.^26^ The saphenous branch of the descending genicular artery (SBDGA) accompanies the saphenous nerve in the distal portion of the adductor canal. Together, they course deep to the posterior border of the sartorius muscle before exiting the adductor canal.^27^

The VAM becomes thickened at its distal edge at the adductor hiatus. With short‐axis ultrasound imaging of the distal thigh, it can be demonstrated as an echogenic thickening, or ‘double contour sign’.^3^, ^28^ Compression of the femoro‐popliteal vessels can occur deep into the distal VAM, resulting in claudication symptoms or leg swelling from venous compression.^29^ Contributors can be due to hypertrophy of the vastus medialis and/or adductor magnus muscles, their associated tendons or the VAM itself. The saphenous nerve is also vulnerable to entrapment at this point, which can result in localised tenderness and pain in the medial knee, radiating to the foot.^3^, ^22^ Surgical decompression may be required to relieve severe neural or vascular symptoms. Immediately distal to the adductor hiatus, the saphenous nerve gives rise to an infrapatellar branch.^30^

### Infrapatellar branch of the saphenous nerve

**Figure 7:**
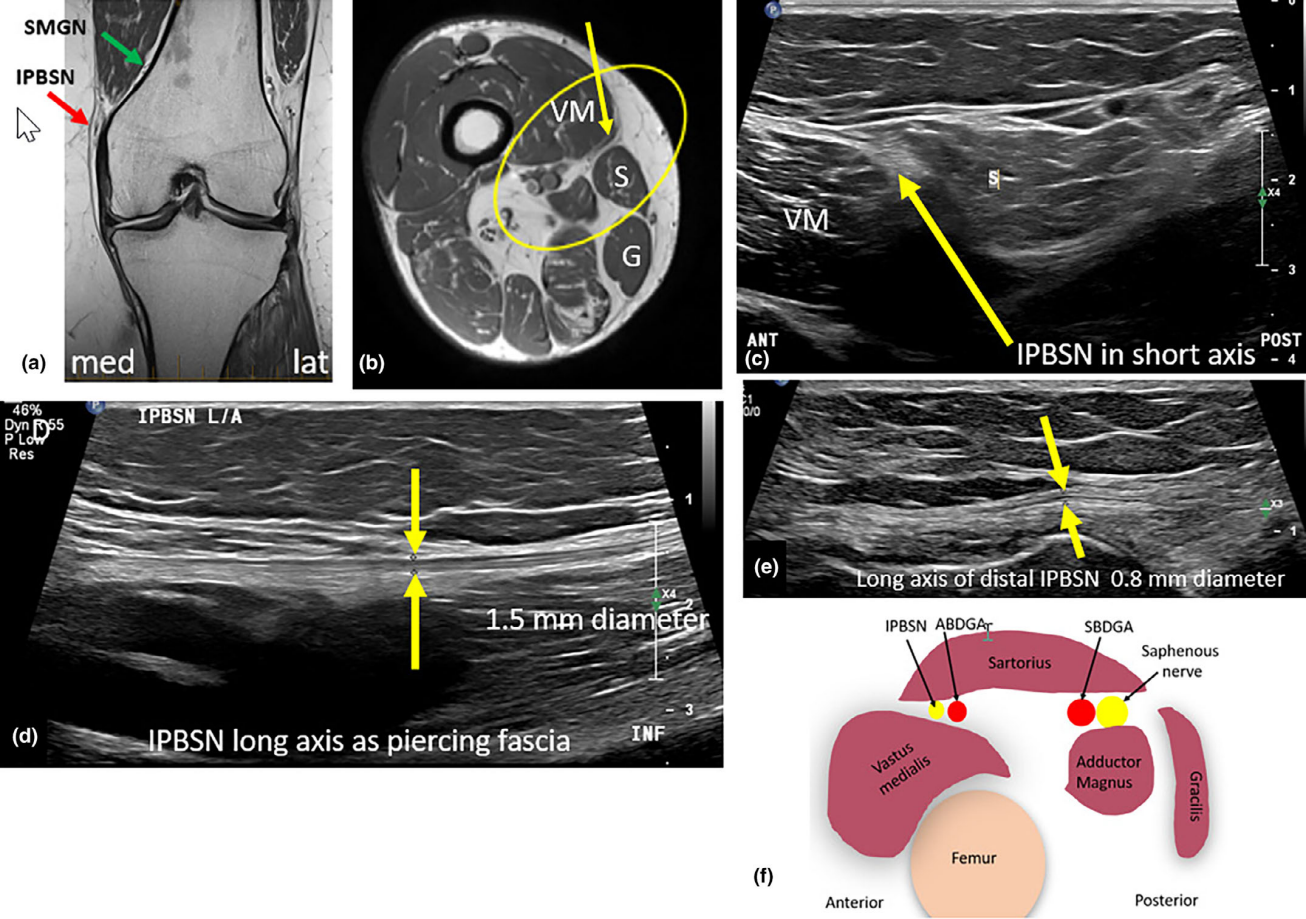
Infrapatellar branch of the saphenous nerve (IPBSN). (a) IPBSN is located superficial to the medial femoral condyle, as shown by the red arrow on the coronal magnetic resonance (MR) image. It is superficial relative to the superomedial genicular nerve (SMGN) indicated by the green arrow. (b) Axial MR image of the right distal thigh. The IPBSN (yellow arrow) typically passes between the sartorius (S) and the vastus medialis (VM) muscles to become superficially located. Yellow oval indicates region of interest. (c) A short‐axis sonographic image of the IPBSN (yellow arrow) between the sartorius and the vastus medialis muscles. (d) Long‐axis image of the IPBSN (yellow arrows) piercing the fascia, moving from deep to superficial. (e) A long‐axis image of the IPBSN (between yellow arrows) in the subcutaneous layer. (f) Diagram showing the typical position of the IPBSN, which courses with the anterior branch of the descending genicular artery (ABDGA) and anterior to the sartorius muscle, whereas the saphenous nerve tends to travel posterior to the sartorius muscle with the saphenous branch of the descending genicular artery (SBDGA). G, gracilis muscle.

The infrapatellar branch of the saphenous nerve (IPBSN) provides sensory innervation to the anteromedial knee, antero‐inferomedial knee joint capsule and anterolateral proximal part of the lower leg.^18^, ^31^ The IPBSN initially follows the path of the articular branch of the descending genicular artery (ABDGA); however, the arterial path of this vessel is variable.^27^, ^32^ Variations in the path of the IPBSN are also described.^30^, ^31^, ^33^, ^34^ Most commonly, the IPBSN travels anteriorly relative to the saphenous nerve with an antero‐inferior trajectory.^33^ The IPBSN traverses the anteromedial aspect of the knee and most commonly courses parallel and anterior to the distal sartorius muscle.^34^ However, it can also variably pass less commonly posterior to the distal sartorius muscle^35^ or penetrate the sartorius muscle^35^ (Figure 7).

The IPBSN travels antero‐inferiorly and superficially to penetrate the deep crural fascia and become located within the subcutaneous tissues of the medial knee. Where the IPBSN pierces the fascia, there is a point of potential entrapment. Once in the subcutaneous tissues, the IPBSN courses inferiorly and anteriorly towards the proximal (femoral) attachment of the medial collateral ligament (MCL) proper, a helpful sonographic landmark.^31^ The IPBSN then travels anteriorly relative to the MCL with an arc‐like course towards the patella apex, lying between this and the tibial tuberosity.^36^ At this point, it gives rise to a variable number of terminal branches, typically between one and three.

The superficial course of the IPBSN makes it susceptible to injury caused by anterior knee trauma or by incisions from open and arthroscopic knee surgeries such as total knee replacement, tendon harvesting of the patellar or hamstring tendons or tibial nail insertions.^3^, ^31^ It can also become entrapped between the sartorius tendon and the medial femoral condyle.^31^ Injury or entrapment to the IPBSN can result in chronic anterior knee pain, dysesthesia and numbness in the anteromedial aspect of the knee.^33^ Changes in the course or structure of the IPBSN can be seen sonographically.^31^ Injury to this nerve can result in a myriad of findings, including diffuse neural thickening, neural transection, or focal neuroma.

### The saphenous nerve in the lower leg

**Figure 8:**
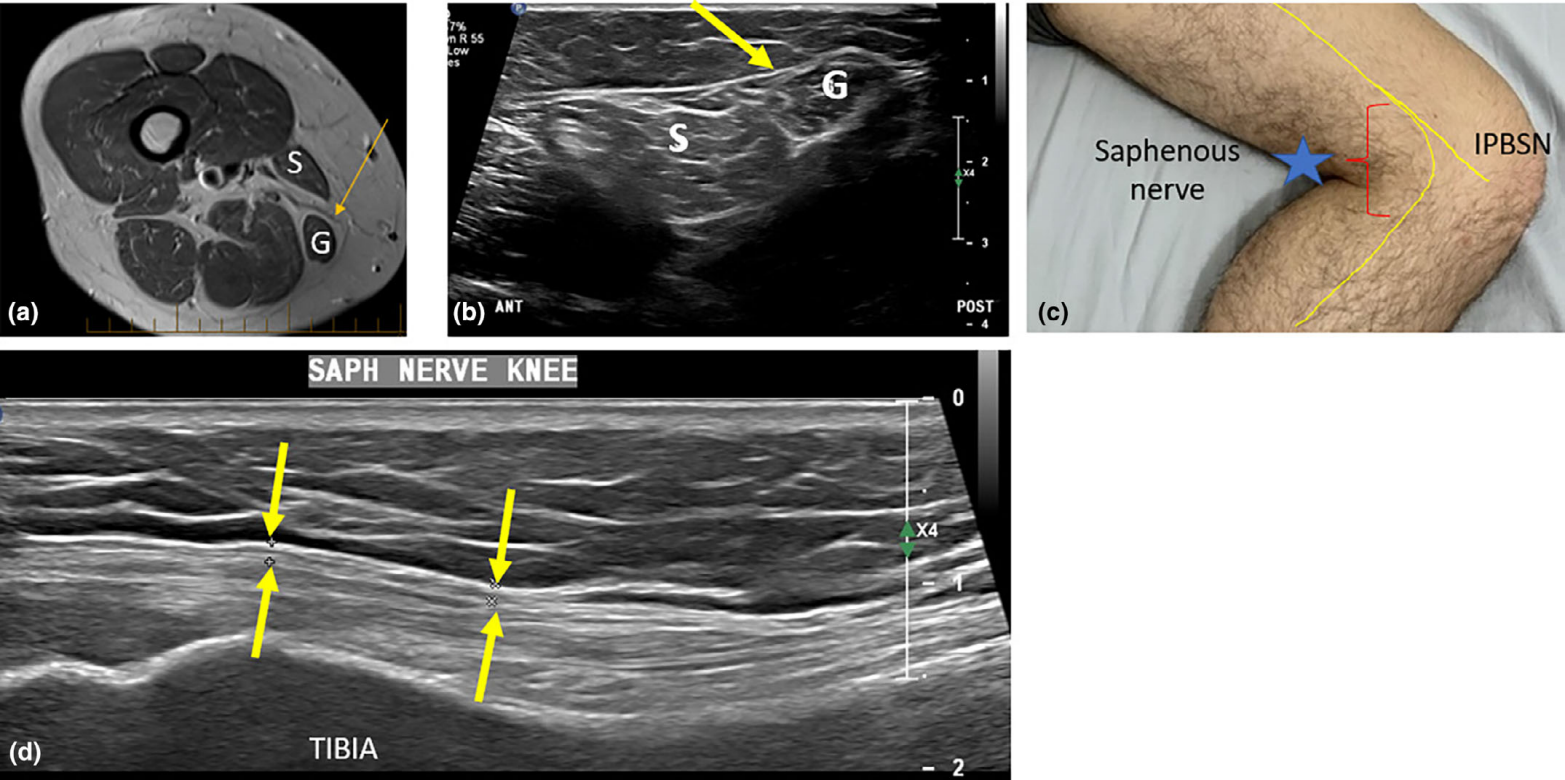
Saphenous nerve at the knee. (a). Axial magnetic resonance (MR) image of the distal thigh, demonstrating the saphenous nerve (orange arrow) passing between the sartorius (S) and gracilis (G) muscles to become superficially located. (b). A short‐axis sonographic image indicating the position of the sartorius and gracilis muscles and the saphenous nerve (yellow arrow) passing between these, where it pierces the crural fascia to become superficially located. (c). The medial knee showing the region in which the saphenous nerve pierces the fascia (red brackets and blue star), posterior to the infrapatellar branch of the saphenous nerve (IPBSN). (d). Sonographic long‐axis image of the saphenous nerve (between yellow arrows) once it has become superficial (approximately 1 mm diameter at this point).

After giving rise to the IPBSN, the saphenous nerve continues distally, posteriorly relative to the IPBSN.^31^ As it is accompanied by the SBDGA, this vessel can be used as a good sonographic vascular landmark for the nerve.^18^ At a variable distance around the knee crease, the saphenous nerve travels from a deep to a superficial location. This occurs between the distal sartorius muscle and the gracilis muscle or tendon. It pierces the fascia to become located within the subcutaneous tissues, where it can potentially become entrapped, and then courses to sit near the great saphenous vein (GSV) (Figure 8).

**Figure 9:**
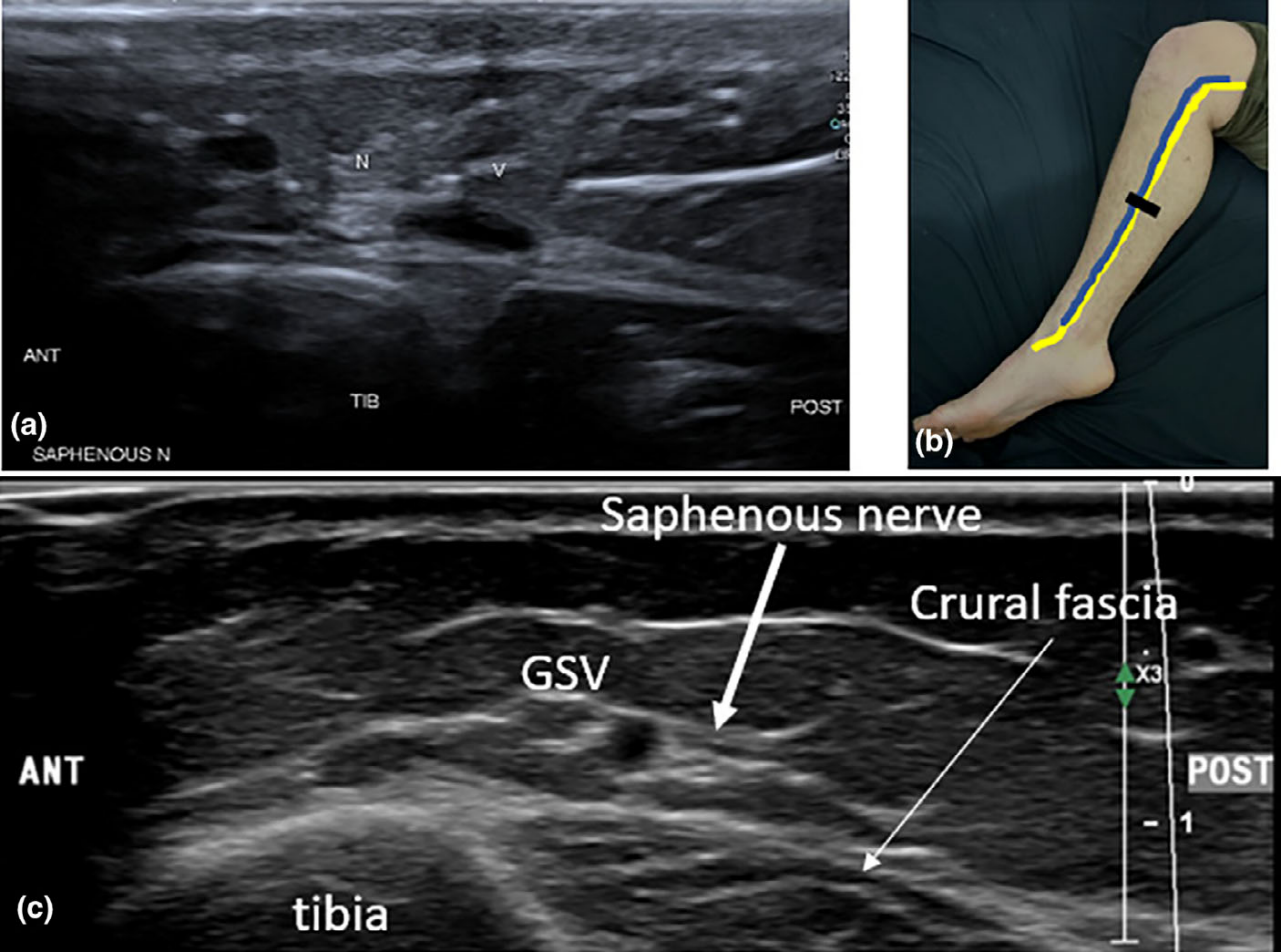
Saphenous nerve in the lower leg. (a) The saphenous nerve (N) can be in variable positions relative to the great saphenous vein (GSV). In this short‐axis sonographic image, the saphenous nerve is anterior to the GSV. It overlies the edge of the tibia (TIB). (b) The position of the saphenous nerve (yellow), which follows the path of the GSV (blue). Transducer position mid‐leg is demonstrated by the black line for short‐axis imaging of the vein and nerve at the mid‐lower leg region, corresponding to image (a). (c) Short‐axis sonographic image demonstrating the saphenous neve (thick white arrow) posterior to the GSV. Both sit superficial to the crural fascia (thin white arrow). ANT, anterior aspect of image; POST, posterior aspect of image.

The superficially located saphenous nerve continues distally along the medial aspect of the lower leg. It can be tracked sonographically in the short axis along the medial tibial border, adjacent to the GSV. It supplies the medial lower leg, ankle and foot^18^ (Figure 9).

The saphenous nerve gives rise to multiple cutaneous branches along its path, which results in it decreasing in size as it travels distally, tapering to approximately 1 mm where it travels anterior to medial malleolus.^18^, ^37^ At the ankle level, the saphenous nerve gives rise to a posterior branch that travels posterior to the medial malleolus; this branch must not be confused with the medial calcaneal nerve. The saphenous nerve and its branches follow the path of the GSV distally and its branches, coursing along the medial and dorsal aspects of the foot and first metatarsophalangeal joint (MTPJ).^38^ Distally, the saphenous nerve provides sensory innervation to the medial aspect of the foot up to the first MTPJ.^39^

**Figure 10:**
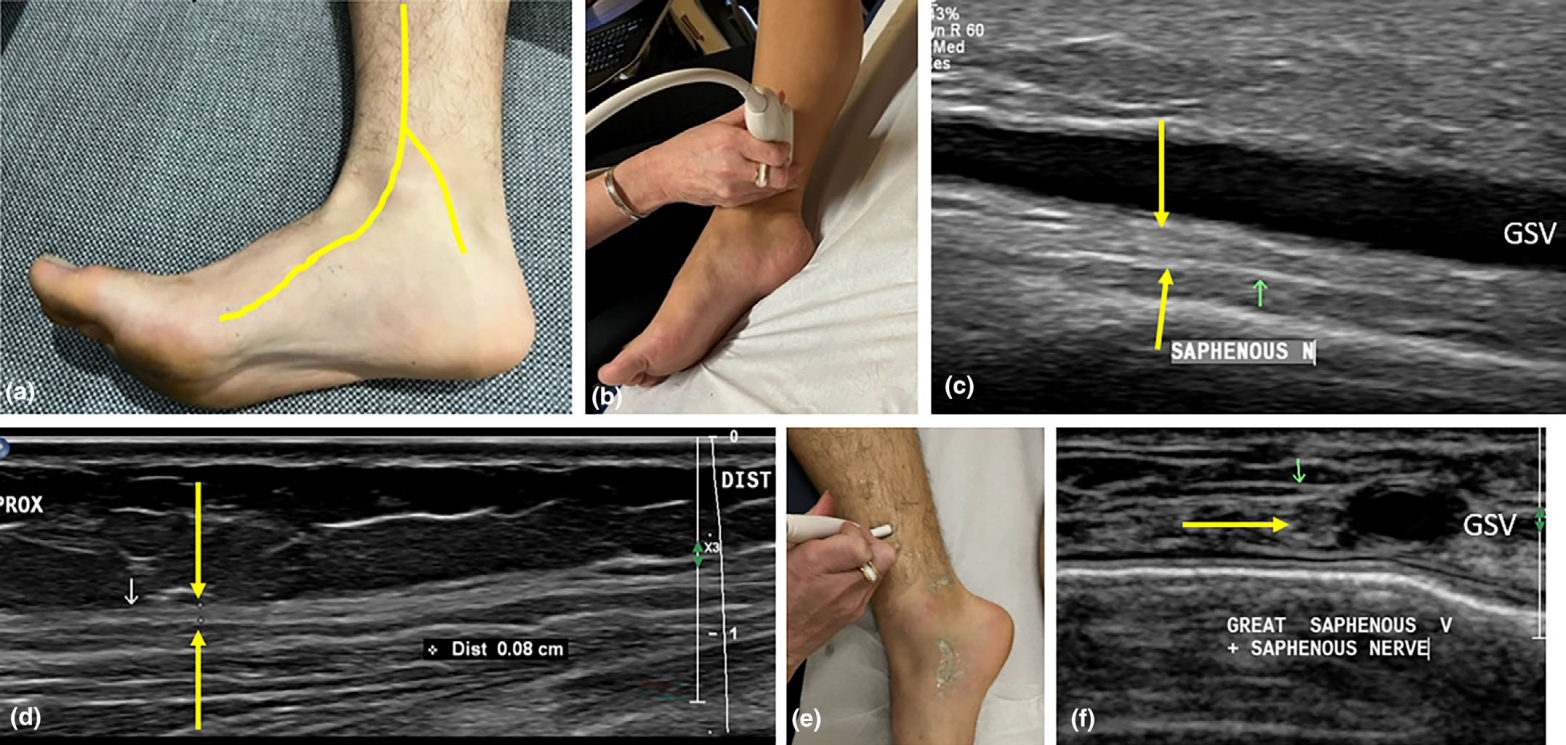
Saphenous nerve at the ankle. (a) The saphenous nerve (yellow) follows the path of the great saphenous vein (GSV), which travels anterior to the medial malleolus. The saphenous nerve gives rise to a posterior branch that courses posterior to the medial malleolus. (b) Transducer placement for long axis imaging of the saphenous nerve. (c) A long‐axis image of the saphenous nerve (yellow and green arrows) at the ankle. The nerve can sit in varying positions relative to the GSV. In this image, the nerve is deep in the GSV. (d) A long‐axis image of the saphenous nerve (outlined by yellow arrows), which has tapered and is 0.8 mm in diameter and sits in the subcutaneous tissues, overlying the deep fascia. (e) Short‐axis transducer positioning for imaging of the saphenous nerve, using a hockey stick transducer. (f) A short‐axis image of the saphenous nerve (outlined by yellow and green arrow) at the ankle adjacent to the GSV and overlying the tibia.

Around the ankle, the saphenous nerve is particularly vulnerable to iatrogenic injury during varicose vein or ankle surgery or due to portals for ankle arthroscopy.^17^ It may also be compromised secondary to fractures of the medial malleolus, ensuing surgical orthopaedic corrections or subsequently entrapped in post‐surgical scar tissue. Sonographically in the lower leg and ankle, the GSV is an excellent landmark to assist visualisation of the saphenous nerve. Long‐ and short‐axis imaging of the nerve can be easily performed. Light transducer pressure should be used to ensure that the GSV is not compressed to mask its visualisation (Figure 10).

## Conclusion

Despite the femoral and saphenous nerves and their branches, including the infrapatellar branch of the saphenous nerve, being within the imaging field during many routine sonographic examinations, these nerves and their branches are commonly overlooked. Awareness and knowledge of nerve paths, their relative anatomy and their sonographic appearances are pertinent to guide nerve blocks and ablations. In addition, the normal and abnormal sonographic appearances of these nerves and their branches must be appreciated to allow associated pathological entities, nerve transections, neuromas and nerve entrapments to be correctly detected. Further research is required to establish normal sonographic measurements of these nerves, which may enhance sonographic discrimination between normal and abnormal‐appearing nerves.

## Conflict of interest

There are no conflicts of interest to declare.

## Author contributions

**Michelle Fenech:** Conceptualization; writing – original draft; writing – review and editing; visualization; project administration; supervision; resources. **Bridie Roche:** Writing – review and editing. **Jerome Boyle:** Writing – review and editing.

## Ethics approval

Human Research ethics committee approval and clearance were not required for the completion of this review paper.
